# Effect of prazosin on diabetic nephropathy patients with positive α1-adrenergic receptor autoantibodies and refractory hypertension

**DOI:** 10.3892/etm.2014.2036

**Published:** 2014-10-27

**Authors:** LIN-SHUANG ZHAO, CHUN-YAN XU

**Affiliations:** 1 Department of Endocrinology, Guangzhou Command Wuhan General Hospital, Wuhan, Hubei 430070, P.R. China; 2 Graduate College, Southern Medical University, Guangzhou, Guangdong 510515, P.R. China

**Keywords:** prazosin, diabetic nephropathy with refractory hypertension, α1 adrenergic receptor, autoantibodies

## Abstract

To investigate the effect of prazosin on patients with diabetic nephropathy (DN), α1-adrenergic receptor (α1-R) autoantibodies and refractory hypertension, a total of 126 patients with DN and hypertension were recruited. The patients were divided into a refractory hypertension group, (n=76) and a non-refractory hypertension group (n=50). The epitope of the second extracellular loop of the α1-R (192–218) was synthesized and an enzyme-linked immunosorbent assay (ELISA) was performed to detect serum autoantibodies. In the group with DN-associated refractory hypertension, the positive rate of autoantibodies against the α1-R was 80.3% (n=61). The 61 patients who were positive for α1-R autoantibodies were randomly divided into a treatment group (n=31) and a control group (n=30). The patients were given drugs at the same dosage and administration, with the exception of prazosin, which was provided only to the patients in the treatment group [1 mg, three times a day (tid)] for a duration of six weeks. Subsequently, prazosin was added (1 mg, tid) to the therapeutic schedule of the patients in the control group and the α1-R autoantibody-negative group for another six weeks. The analysis was carried out on an intention-to-treat basis. The prazosin treatment resulted in significant improvements in hypertension in the treatment group (P<0.05), while there was no marked improvement in the control group. The total effective rate of hypertension improvement was 90.3% in the treatment group, which was higher compared with that of the control group (33.3%). In conclusion, α1-R autoantibodies may play an important role in the pathogenesis of DN with refractory hypertension. Prazosin was demonstrated to be effective and safe in the treatment of DN with refractory hypertension.

## Introduction

Diabetes mellitus (DM) has become a growing global public health issue. The morbidity rate is high and is expected to reach 435 million by 2030 (1). Diabetic nephropathy (DN) in particular is one of the most significant causes of mortality. DN-associated refractory hypertension is common and has a low treatment response rate. Therefore, methods of enhancing the treatment response rate of DN-associated refractory hypertension and reducing cardiovascular and cerebrovascular complications urgently require study. The pathogenesis of DN-associated refractory hypertension is very complicated (2); therefore, studies to increase understanding of the pathogenesis may assist in enhancing the treatment response rate, improving the lives of patients with DN and reducing the cardiovascular and cerebrovascular complications.

G protein-coupled-receptors (GPCRs), which include the α1-adrenergic receptor (α1-R) and angiotensin II type 1 receptor (AT1 receptor), play important roles in the immunologic mechanism of cardio-cerebrovascular complications. They are a set of membrane surface glycoproteins coupled with guanine-binding regulatory proteins. Peptide bonds penetrate the cell membrane seven times, forming a structure where there are segments of the peptide inside and outside the cell with three peptide loops in each. The peptide loops outside the cell receive a signal stimulus, and transmit the extracellular signal to the inside of the cell. GPCR-mediated signals have roles in cardiovascular and visual regulation; they also participate in kidney function and regulate the immune response (3,4). When GPCRs are repeatedly stimulated, autoantibodies are produced through an internal mechanism. The autoantibodies have a pathological activity similar to that of an agonist. Pathological effects are caused by interaction of the autoantibodies with the corresponding receptors, leading to autoimmune reactions and resulting in subsequent tissue damage (5–7).

Early studies revealed that the serum of patients with refractory hypertension and renal damage demonstrated a significantly increased positive rate of the AT1 receptor (7,8). A subsequent study demonstrated that the efficacy of proteinuria reduction in patients with DN who were positive for AT1 receptor autoantibodies and treated with an AT1 receptor antagonist was significantly better than that in patients who tested negative for AT1 receptor autoantibodies; the efficacy differed significantly between patients with different autoantibody status when treated with the same medication (9). Therefore, it may be expected that morbidity due to DN is associated with these receptor autoantibodies. A study has revealed that AT1 receptor antibodies are able to increase the contraction rate of myocardial cells, raise the concentration of free calcium in vascular smooth muscle, and promote hypertension vascular reconstruction (10–12). However, further studies are required to investigate whether refractory hypertension is associated with the participation of α1-R autoantibodies and thus, whether the appliance of α1-R blockers may be effective in alleviating refractory hypertension.

Prazosin is an α1-R antagonist and may be competitively combined with α1-R autoantibodies. The function of prazosin is to selectively block the α1-R postsynaptic membrane of the vascular smooth muscle, relax small arteries and veins, and reduce peripheral resistance, thus lowering blood pressure (13). However, clinical observations have shown that an ideal effect is not achieved in all patients. For further observation, the present study used DN patients with refractory hypertension as subjects. Following testing for the presence of α1-R autoantibodies, targeted treatment with the α1-R blocker prazosin was applied to patients with DN and refractory hypertension who were positive for α1-R autoantibodies.

## Materials and methods

### Patients

A total of 126 patients with DN and ≥level 2 hypertension from the Department of Endocrinology of the Guangzhou Command Wuhan General Hospital (Wuhan, China) were involved in in the current study. The present study was approved by the Human Subjects Committee of Guangzhou Command Wuhan General Hospital and the Southern Medical University. All the subjects gave their written informed consent. The mean ± standard deviation (SD) systolic blood pressure (SBP)/diastolic blood pressure (DBP) was 176.1±11.3/105.4±9.7 mmHg (1 mmHg = 0.133 kPa). Hypertension was diagnosed according to the 1999 ‘World Health Organization (WHO)/International Society of Hypertension (ISH) Hypertension Prevention Guide’ (14). The antihypertensive drug treatment was applied based on the specific circumstances of the patients. The dose and types of drug were increased gradually [captopril 25–50 mg three times per day (tid), nitrendipine 10–20 mg once every 6 h, hydrochlorothiazide 12.5–25 mg once per day, or furosemide 20–40 mg which was intermittently administered orally]. After three months, patients whose blood pressure remained >140/90 mmHg were assigned to the refractory hypertension group, and those whose blood pressure had been controlled by the drugs were placed in the non-refractory hypertension group. The diagnosis of DN was based on the five-phase standard proposed by Mogensen in 1989 (15). Patients with the following characteristics were excluded from the present study: i) other secondary kidney diseases; ii) participation in other clinical studies; iii) DN serum creatinine level >131 μmol/l; iv) DN hyperkalemia; and v) SBP >200 mmHg or DBP >120 mmHg. Among the patients with DN who were involved in the present study, 76 had refractory hypertension (43 males and 33 females with a mean ± SD age of 66.9±9.1 years) and 50 had non-refractory hypertension (26 males and 24 females with an average age of 64.8±7.9 years). All drug applications, with the exception of insulin, were ceased in patients who were included in the current study. All clinical conditions and laboratory examination data were recorded. A 5-ml sample of fasting venous blood was collected from each patient. The plasma was obtained by centrifugation and stored at −40°C for later use.

### Solid phase peptide synthesis

An automatic peptide synthesizer (PSSM-8; Shimadzu, Kyoto, Japan) was used to synthesize peptide fragments targeting the epitopes of the second extracellular loop of α1-R (192–218; G-W-K-E-P-V-P-P-D-E-R-F-C-G-I-T-E-E-A-G-Q-A-V-F-S-S-V) (16). The purity of the synthetic peptides, analyzed by high performance liquid chromatography, was >95%.

### Detection of autoantibodies by enzyme-linked immunosorbent assay (ELISA)

The autoantibodies were detected according to a previous method (16). Blank, positive and negative controls were set up. The optical density (OD) of the blank control was set at zero in order to ensure the reliability of the results. Using serum from healthy individuals as a negative control and a reference point, the positive control was established with an OD two-fold higher than the reference OD. Testing positive for autoantibodies was defined as follows: OD value of the sample - OD value of the blank control/OD value of the negative control - OD value of the blank control >2.1.

### Urinary albumin excretion rate (UAER) determination

Urine was collected for 24 h and benzoic acid was used for its preservation. The total volume was recorded and the 24-h urine protein level was determined by ELISA (Shanghai Baoman Biotechnology, Shanghai, China).

### Methods of medication application and grouping

Patients with refractory hypertension who were positive for α1-R autoantibodies were randomly divided into the treatment group (group A) and control group (group B). Prazosin (1 mg tid) was administered to the treatment group only. The following medications were administered to the two groups: Captopril (25–50 mg tid), nitrendipine (10–20 mg once every 6 h), hydrochlorothiazide (12.5–25 mg once per day) and enteric-coated aspirin (100 mg once per day). The antihypertensive efficacy of prazosin in groups A and B was compared. Following the first six weeks of the study, patients with refractory hypertension who tested negative (group C) and positive (group D) for α1-R autoantibodies were administered the same medication as group A. The antihypertensive efficacy of prazosin in groups C and D was compared.

### Blood pressure observation

Blood pressure was measured twice per day (at 07:00 and 22:00) with a standard cuff and mercury sphygmomanometer, with the patients seated for 10 min prior to measurement. SBP and DBP were recorded. During the treatment period, patients were permitted to carry out normal daily activities and work, but prevented from doing intensive exercise.

### Evaluation of the antihypertensive efficacy

Evaluation was based on the criteria for drug efficacy revised by the National Meeting of Cardiovascular Diseases in April 1979 (17): i) markedly effective: DBP is lowered by ≥10 mmHg (1.33 kPa) and SBP is decreased to normal or by >20 mmHg (2.67 kPa); ii) effective: although the DBP is lowered <10 mmHg, the SBP reaches normal levels. In the case of hypertension during the systolic period, a >30 mmHg (4 kPa) reduction of SBP is considered effective; iii) not effective: the reduction in blood pressure does not reach the above standard.

### Statistical analysis

The data are presented as mean ± SD and were processed using SPSS software, version 10.0 (SPSS, Inc., Chicago, IL, USA). The independent samples t-test was used to analyze the measurement data and the χ^2^ test was used for the enumeration data. P<0.05 was considered to indicate a statistically significant difference.

## Results

### General clinical data characteristics

Statistically significant differences were observed only in the blood pressure and UAER between the DN patients with and without refractory hypertension (P<0.05; Table I).

### Comparison of α1-R autoantibodies between the DN refractory and non-refractory hypertension groups

The number of positive cases and rate of the α1-R in the DN refractory hypertension group were significantly higher than those in the DN non-refractory group (P<0.01). This suggests that DN refractory hypertension may be associated with the pathogenic involvement of α1-R autoantibodies (Table II).

### Comparison of the antihypertensive efficacy between the treatment and control groups of patients with DN, refractory hypertension and a positive anti-α1-R status (groups A and B)

The blood pressure of group A dropped markedly following one week of treatment and decreased to 132±5/81±5 mmHg after two weeks. Reexamination following six weeks of treatment revealed a further reduction to 129±5/78±4 mmHg. However, group B experienced no improvement in blood pressure following one, two and six weeks of treatment. The comparison of the two groups revealed statistically significant differences between the blood pressure measurements (P<0.05 at one and two weeks and P<0.01 at six weeks; Table III). The results indicate that prazosin is markedly effective as an antihypertension treatment for refractory hypertension.

### General evaluation of the clinical antihypertensive efficacy between the treatment and control groups with DN, refractory hypertension and a positive anti-α1-R antibody status (groups A and B)

The efficacy in the treatment group was significantly more improved than that in the control group. The difference between the two groups was statistically significant (P<0.05; Table IV).

### Comparison of the antihypertensive efficacy of prazosin between patients with negative and positive α1-receptor autoantibody status in the DN refractory hypertension groups (groups C and D)

Following the administration of prazosin for one week, a significant improvement in blood pressure was observed in group D. Two weeks of continuous application lowered the blood pressure to 133±5/79±5 mmHg, and after six weeks, it was decreased to 123±7/77±3 mmHg. However, there was no notable improvement in group C following one week of treatment, and measurements at two and six weeks following treatment revealed that the blood pressure was not controlled within the normal range. The differences in blood pressure between the two groups were statistically significant (P<0.05 at one and two weeks and P<0.01 at six weeks; Table V).

### Clinical observation of the side-effects of prazosin

In the group of 31 patients with DN and refractory hypertension that was treated with prazosin for 12 weeks (group A), one patient had orthostatic hypotension, which was associated with a poor diet, and one patient had itchy skin. Upon applying calamine lotion, the itchiness disappeared and prazosin application was continued. In the same group, two male cases experienced occasional difficulty in urinating as a result of combined prostatic hyperplasia. Following the application of prazosin, urination improved. No palpitations or dizziness were observed.

## Discussion

GPCRs are a group of membrane surface glycoproteins that combine with guanylate to regulate protein coupling. The α1-R receptor belongs to the GPCR family (18). The GPCR structure is regular in form and the ligands have a high specificity to their receptors (19). This specificity forms the foundation for the regulation of cell signaling and also presents a target for drug treatment (20). Receptor blockers are used to interfere with the receptors in order to block pathological effects (21,22) and are therefore important in the protection of target organs.

The results of the present study revealed that of the 126 patients with DN hypertension, 76 were refractory which was a significant majority of the patients; the remaining 50 were non-refractory. The UAER of the refractory patients was notably higher than that of the non-refractory patients. The difference between the two groups was statistically significant. To further analyze the cause of the DN-associated refractory hypertension, the pathogenic mechanisms at the receptor level were studied. Firstly, a serum test of α1-R autoantibodies was conducted for all patients in the study. The α1-R autoantibody positive rate of 80.3% (61/76) in the group with DN refractory hypertension was significantly higher than the positive rate of 12.0% (6/50) in the group with non-refractory hypertension (P<0.01). This suggests that DN refractory hypertension may be associated with the pathogenic involvement of this receptor, and that the stimulating function of the anti-α1-R autoantibodies may play a role in the pathogenesis of hypertension. It has been reported that anti-α1-R autoantibodies exist in the serum of patients with malignant hypertension. These autoantibodies may also be obtained from rats immunized by synthetic polypeptides targeting the extracellular second loop of α1-R (192–218 amino acid residues) (23). Furthermore, Iwata *et al* have demonstrated that a dose-dependent positive chronotropic effect on the myocardial cells of newborn mice and activity similar to that of a receptor agonist may be blocked by the α1-R antagonist prazosin (24). The involvement of anti-α1-R autoantibodies in the pathogenic process of hypertension may be one of the main causes of refractory hypertension. A study by Fu *et al* investigated α1-R autoantibodies in patients with malignant hypertension (25). Consistent with the results of the current study, Fu *et al* observed that the binding of the antibody and its receptor may produce an effect similar to that of a receptor agonist. This may play a significant role in the pathogenesis of hypertension and its complications.

To further investigate the clinical antihypertensive efficacy, a subgroup analysis was carried out on the 61 cases with DN-associated refractory hypertension who tested positive for α1-R autoantibodies. Prazosin was initially only applied to the treatment group, resulting in a significantly higher antihypertensive efficacy in the treatment group compared with that in the control group. Notably, clinical evaluation of the patients with DN-associated refractory hypertension who tested positive for anti-α1-R antibodies revealed that the general efficacy rate in the prazosin treatment group was 90.3% compared with 33.3% in the control group, taking into account statistical differences.

In order to analyze the difference in the efficacy of prazosin between the positive and negative α1-R autoantibody groups, the same drug treatment was applied to the two groups. The results demonstrated that the efficacy was higher in the α1-R autoantibody-positive group, thus supporting the hypothesis that antihypertensive efficacy differs between patients who are positive for α1-R antibodies and those who are negative, and providing an explanation as to why the same treatment may result in differing levels of efficacy. For example, with the same clinical drug application, the antihypertensive efficacy may be ideal for some patients but not for others. As a consequence, the targeting of therapy according to the individual situation of a patient may prevent the wasting of medical resources, misjudgment of the timing for treatment and avoid harm to organs caused by a continuously low treatment response rate. Analyzing the pathogenic mechanisms at the receptor level and then targeting the treatment according to the specific situation of the patient may be one of the key measures for enhancing the response rate to antihypertensive treatment.

The pathogenic mechanisms of hypertension are extremely complicated (26,27) and include: insulin resistance, renal sodium and water retention, and the activation of the sympathetic nervous system and the renin angiotensin aldosterone system (RAAS). It is often difficult to control blood pressure to a level that complies with the standard required, especially in elderly patients with DN-associated hypertension. Long-term hypertension may result in systemic small vessel disease, the proliferation and fibrosis of the medial smooth muscle cells of small arteries, and the thickening and narrowing of the vascular walls, causing blood shortages and subsequent damage to important target organs, including the heart, brain and kidney tissues (28). Therefore, the aim of the present study was to aid the selection of appropriate antihypertensive drugs and provide solutions to the current challenges faced by clinical doctors. However, due to the intricacy of the pathogenic mechanisms of hypertension, further study is required.

The mechanism of action of prazosin is the selective blocking of the postsynaptic membrane α1-R of the vascular smooth muscle, the relaxation of small arteries and veins, and the reduction of peripheral resistance. There may be a slight increase in cardiac output. Long-term application of prazosin may improve lipid metabolism, lower total cholesterol, triglycerides and low-density lipoprotein cholesterol levels, and raise high-density lipoprotein cholesterol levels, with a positive effect on glucose metabolism (29). It may also reduce the difficulties in urination faced by elderly people due to prostatic hyperplasia (30). The α1-R blocker prazosin may also safely and effectively lower blood pressure; however, for elderly patients, orthostatic hypotension should be prevented. Prazosin treatment is also advantageous to patients with dyslipidemia or an abnormal glucose metabolism.

In conclusion, detecting α1-R autoantibodies in the serum, considering the pathogenic mechanism at the receptor level and applying target treatment with a receptor antagonist accordingly for patients with DN-associated refractory hypertension is of clinical significance for enhancing the antihypertension treatment response rate.

## Figures and Tables

**Table I:** General clinical data characteristics of the patients with DN and hypertension.

Hypertension group	N	Gender (M/F)	Age (years)	Course (years)	SBP/DBP (mmHg)	HR (beats/min)	BUN (mmol/l)	Cr (mmol/l)	UAER (mmol/l)
Refractory	76	43/33	66±9	11±4	176±11/101±7^a^	89±9	9.9±1.2	111.1±7.6	317±29^a^
Non-refractory	50	26/24	5±11	10±5	142±15/88±9	86±11	8.9±1.6	113.9±9.2	206±31

DN, diabetic nephropathy; SBP, systolic blood pressure; DBP, diastolic blood pressure; HR, heart rate; BUN, blood urea nitrogen; Cr, serum creatinine; UAER, urinary albumin excretion rate. Data are presented as means ± standard deviations.

a P<0.05 vs. the DN non-refractory hypertension group.

**Table II:** Comparison of test results for anti-α1 receptor autoantibodies in patients with DN and hypertension.

Hypertension group	N	α1-receptor positive cases	Positive rate (%)	α1-receptor negative cases	Negative rate (%)
Refractory	76	61^a^	80.3^a^	15^a^	19.7^a^
Non-refractory	50	6	12.0	44	88.0

DN, diabetic nephropathy.

a P<0.01 vs. the DN non-refractory hypertension group.

**Table III:** Comparison of anti-hypertensive efficacies between the treatment and control groups.

		SBP/DBP (mmHg)			
		
Group	N	Prior to treatment	1 week after treatment	2 weeks after treatment	6 weeks after treatment
Treatment	31	176±11/101±7	151±6/91±5^a^	132±5/81±5^a^	129±5/78±4^b^
Control	30	175±12/105±5	171±10/99±5	169±5/91±5	161±5/90±5

SBP, systolic blood pressure; DBP, diastolic blood pressure.

a P<0.05,

b P<0.01 vs. the control group.

**Table IV:** Comparison of clinical effectiveness between the treatment and control groups.

Group	N	Not effective, n (%)	Effective, n (%)	Markedly effective, n (%)	General effectiveness rate, %
Treatment	31	3 (9.7)^b^	7 (22.6)^a^	21 (67.7)^b^	90.3^b^
Control	30	20 (66.7)	4 (13.3)	6 (20.0)	33.3
χ^2^-value		21.339	9.258	19.331	31.627
P-value		0.0023	0.039	0.00269	0.0005

a P<0.05

b P<0.01 vs. the control group.

**Table V:** Comparison of the antihypertensive efficacy of prazosin between the α1-adrenergic receptor autoantibody negative and positive groups of patients with DN-associated refractory hypertension.

		SBP/DBP (mmHg)			
		
Group	N	Prior to treatment	1 week after treatment	2 weeks after treatment	6 weeks after treatment
Negative	15	163±7/101±5	160±7/97±4	169±5/91±5	159±5/90±4
Positive	30	161±9/100±6	147±5/93±5^a^	133±5/79±4^a^	123±5/77±3^b^

DN, diabetic nephropathy; SBP, systolic blood pressure; DBP, diastolic blood pressure.

a P<0.05,

b P<0.01 vs. the negative group.

Data are presented as means ± standard deviations.
